# Full Band Spectra Analysis of Gait Acceleration Signals for Peripheral Arterial Disease Patients

**DOI:** 10.3389/fphys.2018.01061

**Published:** 2018-08-07

**Authors:** Mihaela I. Chidean, Óscar Barquero-Pérez, Rebeca Goya-Esteban, Alberto Sánchez Sixto, Blanca de la Cruz Torres, Jose Naranjo Orellana, Elena Sarabia Cachadiña, Antonio J. Caamaño

**Affiliations:** ^1^Signal Theory and Communications Department, University Rey Juan Carlos, Fuenlabrada, Spain; ^2^Physical Activity and Sports Department, University CEU San Pablo, Seville, Spain; ^3^Physiotherapy Department, University of Seville, Seville, Spain; ^4^Sports and Computer Science Department, University Pablo de Olavide, Seville, Spain

**Keywords:** peripheral arterial disease, gait, full spectral, fundamental frequency, spectral envelope

## Abstract

Peripheral arterial disease (PAD) is an artherosclerotic occlusive disorder of distal arteries, which can give rise to the intermittent claudication (IC) phenomenon, i.e., limb pain and necessity to stop. PAD patients with IC have altered their gait, increasing the fall risk. Several gait analysis works have studied acceleration signals (from sensors) to characterize the gait. One common technique is spectral analysis. However, this approach mainly uses dominant frequency (*f*_*d*_) to characterize gait patterns, and in a narrow spectral band, disregarding the full spectra information. We propose to use a full band spectral analysis (up to 15 Hz) and the fundamental frequency (*f*_0_) in order to completely characterize gait for both control subjects and PAD patients. Acceleration gait signals were recorded using an acquisition equipment consisting of four wireless sensor nodes located at ankle and hip height on both sides. Subjects had to walk, free-fashion, up to 10 min. The analysis of the periodicity of the gait acceleration signals, showed that *f*_0_ is statistically higher (*p* < 0.05) in control subjects (0.9743 ± 0.0716) than in PAD patients (0.8748 ± 0.0438). Moreover, the spectral envelope showed that, in controls, the power spectral density distribution is higher than in PAD patients, and that the power concentration is hither around the *f*_*d*_. In conclusion, full spectra analysis allowed to better characterize gait in PAD patients than classical spectral analysis. It allowed to better discriminate PAD patients and control subjects, and it also showed promising results to assess severity of PAD.

## 1. Introduction

Peripheral Arterial Disease (PAD) is an artherosclerotic occlusive disorder of arteries distal to the aortic bifurcation (Ramos et al., 2009). Due to the arterial occlusion, lower limb muscles do not receive the oxygen required while exercising, provoking pain, and the necessity of stop walking. This phenomenon is called intermittent claudication (IC) and it may result in limitations in daily physical activities and impairments in health-related quality of life (Feinglass et al., 1996; Crowther et al., 2007; Celis et al., 2009). Atherosclerosis, and thereby PAD, are especially found in elderly and it is associated to diabetes mellitus and other cardiovascular risk factors such as hypertension, high body mass index, and dyslipidemia (Diehm et al., 2009; Ramos et al., 2009). Smokers increase also the possibilities of developing PAD (Ramos et al., 2009). PAD is asymptomatic in the first stages of the disease and, in more advanced stages, PAD turns symptomatic appearing IC (Diehm et al., 2009). Therefore, claudicating patients tend to reduce their mobility due to pain (Celis et al., 2009). Moreover, the loss of work capacity affect not only to the ischemic limb but also to the healthy one (Wurdeman et al., 2012).

PAD patients have altered their gait characteristics, showing higher levels of gait variability compared to healthy matched controls (Myers et al., 2009, 2011). In some studies, gait analysis is used to study differences between PAD patients and healthy subjects, and eventually, to identify differences among PAD patients at different stages (Chen et al., 2008; Myers et al., 2009). Ultimately, gait analysis can be used to assess treatment influences on altered gait (Huisinga et al., 2010).

Falling is one of the main consequences of the PAD, therefore, the development of methods to predict fall risk in PAD patients is of great importance. Several studies have assessed gait using spectral characteristics of acceleration signals to evaluate fall risk (Antonsson and Mann, 1985; Yack and Berger, 1993; Weiss et al., 2011, 2013). However, the usual approach is mainly focused on the information provided by the dominant frequency (*f*_*d*_) of the gait, and discards full spectra information. We propose to characterize gait using the full spectra analysis, namely, using the fundamental frequency *f*_0_ to represent the gait period, and the harmonic structure to provide a complete description of the gait characteristics. We used an acquisition systems of four sensor nodes placed at ankle and hip height, developed by our group, to record the acceleration signals during gait (Chidean et al., 2017).

The structure of the manuscript is as follows. In section 2, the data acquisition equipment is described. In section 3 the proposed method and new indices are explained. In section 4, the data set and analysis setup is detailed. Results are presented in section 5, while discussion and conclusions are presented in section 6.

## 2. Gait data acquisition equipment

**Figure 1:**
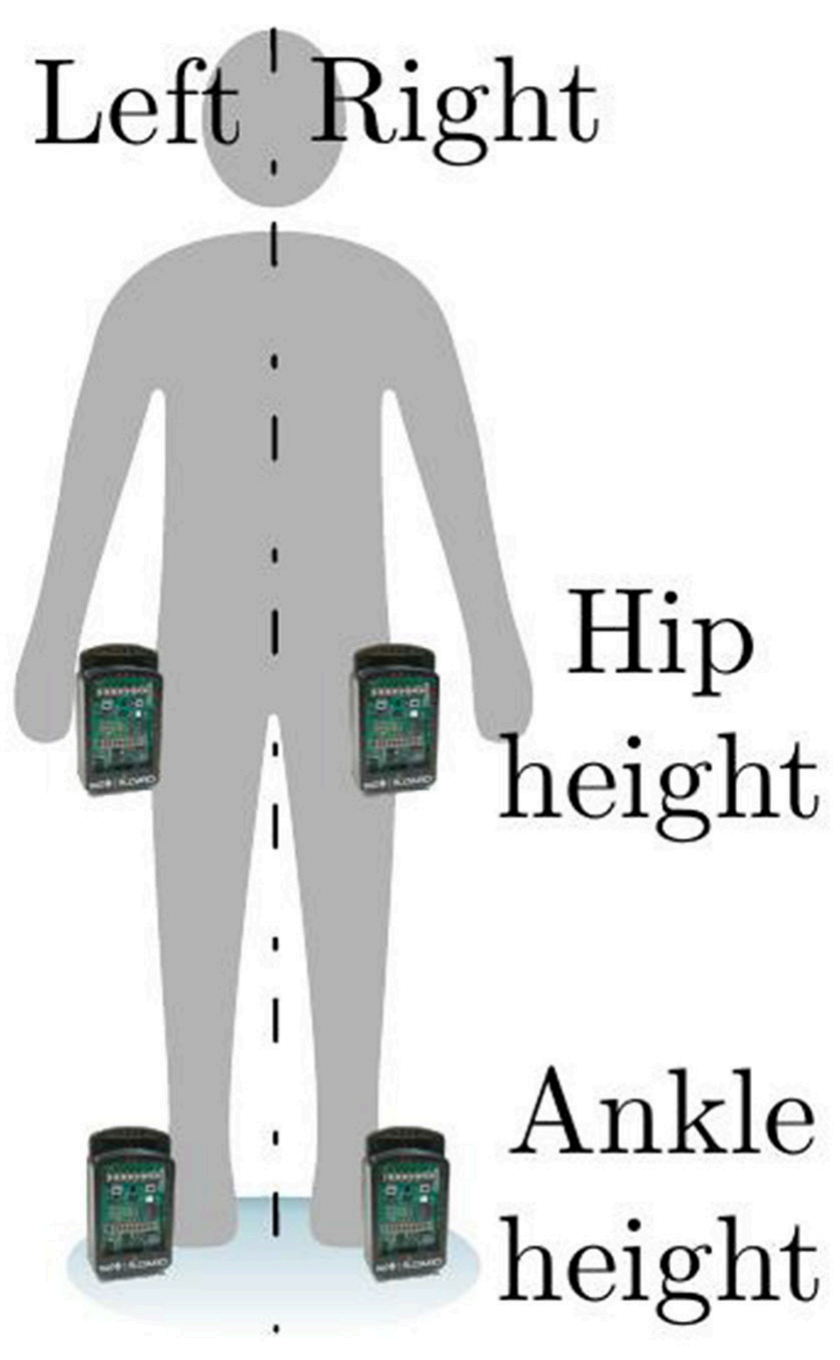
Localization of the measurement devices and spatial axes orientation.

We propose to use a non-invasive, small equipment to acquire gait data based on a wireless sensor network. This system does not bother the subject and enhances spontaneous and natural movement. It consists of four devices that measure the tri-axial ±8 g acceleration and that are located at hip and ankle height (Chidean et al., 2017). Figure 1 shows the localization of these devices and their spatial orientation with respect to the 3D Cartesian axes and the walking direction. Acceleration signals are measured with a 50 Hz sampling frequency.

In this work we use this gait data acquisition equipment as it allows us to measure the natural gait, aiming to assess differences on spontaneous gait between healthy subjects and PAD patients. We discarded specialized equipment, such treadmill (Giandolini et al., 2014; Sloot et al., 2014a), in-floor or insole devices (Hausdorff et al., 2000; Yogev et al., 2007), since the gait is conditioned and it can also be dangerous in PAD patients, thus increasing the risk of falling (Hausdorff, 2007; Sloot et al., 2014a,b).

## 3. Methods

### 3.1. Classical gait spectral analysis

Spectral analysis of acceleration gait signals has been proved to be a valuable tool assessing gait patterns in elderly and falling subjects (Yack and Berger, 1993; Weiss et al., 2013). Spectral analysis characterizes the quantity and quality of the gait pattern. Usually, frequency domain indices are based on the characteristics of the *dominant frequency* peak, *f*_*d*_, in the power spectral density (PSD). Common indices computed from the PSD are, *f*_*d*_ (Hz), the bandwidth, *bw*_*f*_*d*__ (Hz). These indices aim to characterize periodicity and variability of acceleration gait signals (Yack and Berger, 1993; Weiss et al., 2011, 2013). Spectral analysis is commonly restricted to the frequency band of [1−3] Hz, considered the band of interest, where the dominant frequency lies (Moore et al., 2008).

### 3.2. Full spectra analysis

**Figure 2:**
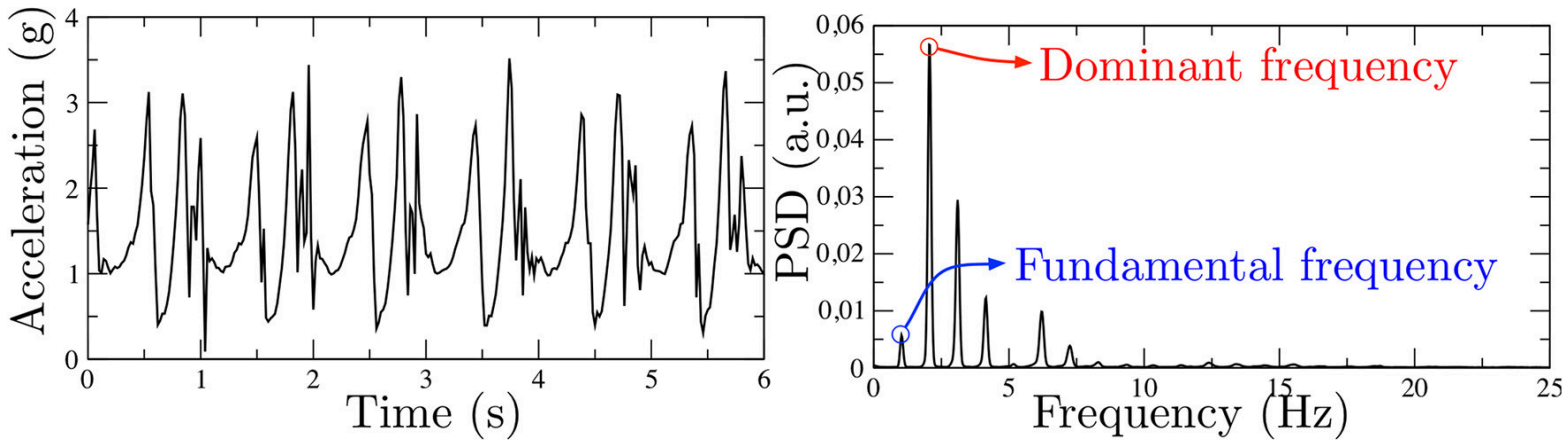
Real gait acceleration signal in time domain **(Left)** and frequency domain **(Right)**, where a clear harmonic structure can be observed.

Though highly informative, parameters yielded by classical spectral analysis could give an incomplete spectral description of gait acceleration signals. The implicit assumption in classical analysis is that acceleration signal are almost-pure sinusoidal signals, since most of the energy is concentrated around the main peak, which, under this assumption, corresponds to the inverse of the period. However, in most cases, acceleration signals are better modeled as pseudo-periodic signals with a clear structure of harmonics, which model the shape of the pattern repeated along the gait. Figure 2 represents an acceleration signal and its PSD, where the harmonic structure is clear.

We propose a *full spectral* analysis, which considers a wider frequency band, [0.3−15] Hz. Our approach includes the locomotor frequency band (Moore et al., 2008; Weiss et al., 2013), allowing for complete gait characterization. We propose to analyze the periodicity and variability of acceleration gait signals with the following parameters computed on the PSD.

#### Periodicity characterization

Acceleration gait signals are quasi-periodic, i.e., there is a pattern that is repeated in regular intervals. Periodicity can be estimated in the frequency domain using the **fundamental frequency**, *f*_0_ (Hz), which, in pseudo-periodic signals, represents the inverse of the period. Therefore, we propose to use *f*_0_ as a parameter to estimate the periodicity of the gait. *f*_0_ should correspond to the first peak on the PSD. In some cases, depending on harmonic structure, *f*_0_ may coincide with the dominant frequency. To Estimate *f*_0_ is usually challenging, and some preprocessing is needed to obtain reliable estimations. We propose the following preprocessing, inspired by spectral analysis in atrial fibrillation (Botteron and Smith, 1995; Barquero-Pérez et al., 2010): (1) First the original signal is high-pass filtered with a cut frequency of 20 Hz, then, (2) the signal is rectified, and finally, (3) low-pass filtered with a cut frequency 1.5 Hz (Ng and Goldberger, 2007; Barquero-Pérez et al., 2012). This preprocessing aims to obtain a new signal where the dominant frequency and *f*_0_ coincide, and can be easily estimate using the PSD of resulting preprocessed signal.

#### Variability characterization

Variability is related to how the power is distributed among the different spectral peaks (*f*_0_ and harmonics). We propose two different parameters, *regularity index*, *ri* defined as the ratio of the power in the *bw*_*f*_*d*__ and the total power in the band of interest [0.3−15] Hz. To give a complete characterization of the harmonic structure, we propose to compute the spectral envelope, which for a purely periodic signal is given by the Fourier Transform of a single cycle. For an almost-periodic signal, the spectral envelope is still (roughly) related to the averaged Fourier Transforms of consecutive cycles. The spectral envelope of the acceleration signal was computed by means of Linear Predictive Coding (LPC) analysis. Using LPC analysis we assume that an acceleration signal sample *s*[*n*] can be estimated as a linear combination of *p* past samples:

(1) $$\begin{array}{l} ŝ\left[n\right]=\overset{p}{\underset{k=1}{\sum }}a_{k}s\left[n-k\right] \end{array}$$

We use this assumption since we seek to emphasize the repetitive structure inherently present in the acceleration signal. The LPC coefficients *a*_*k*_ are determined by minimizing the squared error of the predicted signal (ŝ[*n*]) and the actual signal. Therefore, *a*_*k*_ are the coefficients of the time-varying digital filter whose steady-state function in of the form:

(2) $$\begin{array}{c} H(z)=\frac{G}{1-\sum_{k=1}^{p}a_{k}z^{-k}} \end{array}$$

Where *G* is the gain parameter. The spectral envelope of the gait signal is represented, in a compressed form, as the magnitude of the frequency response of this LP filter.

In the context of our study, we will use *f*_0_ to assess changes in gait periodicity (*inter-step*) due to PAD. Additionally, we will use regularity index *r*_*i*_ and the spectral envelope to assess *intra-step* changes due to PAD. We will compare the results with dominant frequency *f*_*d*_ characterization. Table 1 summarizes the definition of these parameters^1^.

**Table 1:** Parameter summary.

**Parameter**	**Meaning**
*f*_*d*_	Dominant frequency
*bw*_*f*_*d*__	Bandwidth around the dominant frequency
*f*_0_	Fundamental frequency
*bw*_*f*_0__	Bandwidth around the fundamental frequency
*ri*	Regularity index
*Spectral envelope*	

## 4. Data base and analysis setup

The study was carried out following the principles of the Helsinki Declaration and it was approved by the Local Ethics Committee. In this section, firstly, the acquired database is detailed, and secondly, the statistical analysis setup is described.

### 4.1. Data base

Gait data from 8 PAD patients and 10 controls were collected using the acquisition equipment described in section 2. All participants were aged between 50 and 68 years. The average age (± standard deviation) was 62.32(±3.85) and 60.20(±4.98) for the eight patients and the 10 controls, respectively.

**Figure 3:**
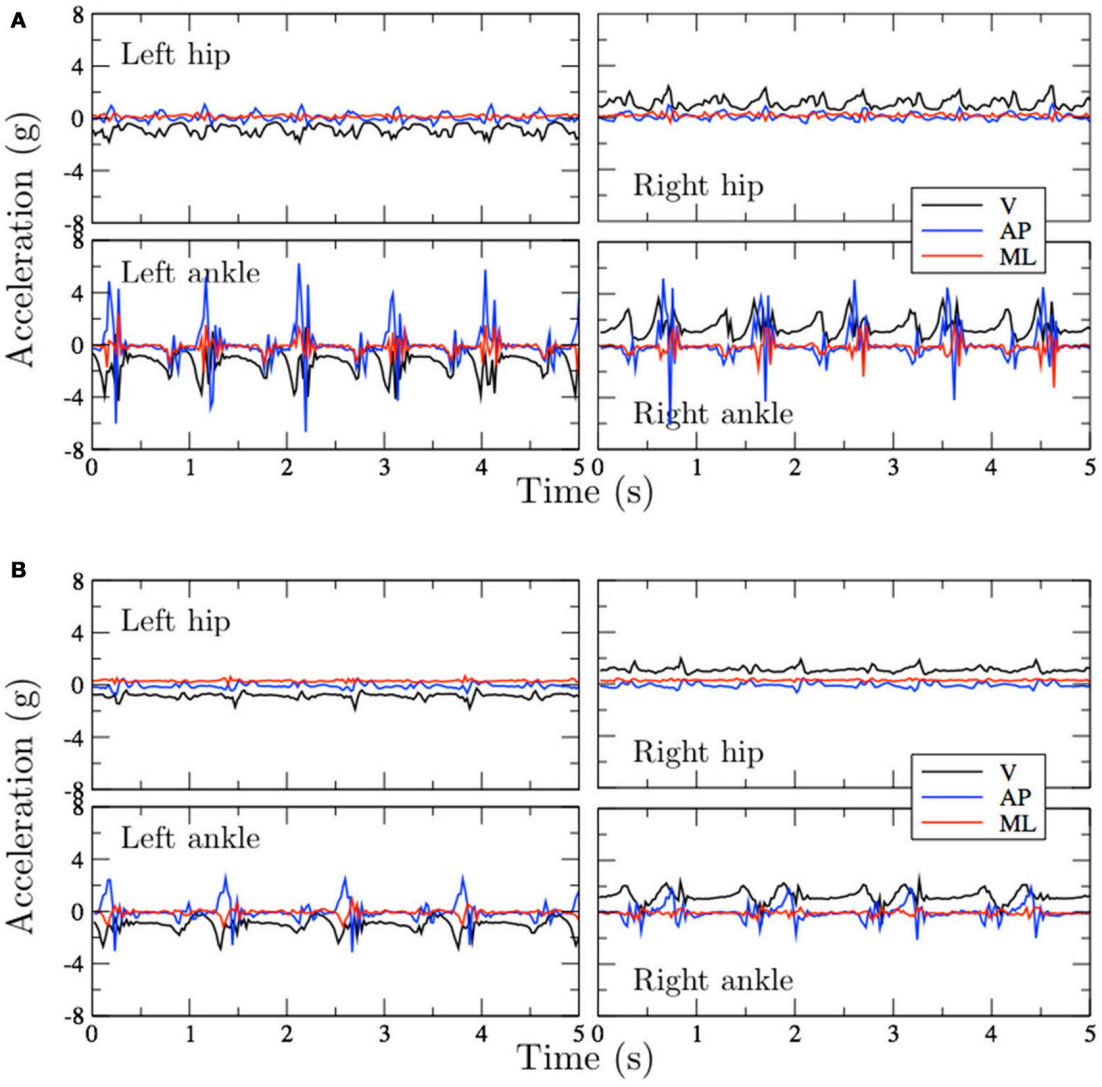
Acceleration signals obtained from experiments with two subjects walking on an indoor corridor. **(A)** Control (65 years). **(B)** PAD patient (68 years).

Figure 3 shows raw acceleration signals, obtained from independent experiments. This figure includes data from the four sensor locations and for the three spatial axes.

The inclusion criteria for controls were: not suffering from cardiovascular disease, not following any medical treatment and having an Ankle Brachial Index (ABI) >1. PAD patients were recruited from two Hospitals in Sevilla (Spain). The inclusion criterion for PAD patients was to be referred by the Vascular Surgery Service. The patient history included the diagnosis of PAD without surgery and an ABI < 0.9 (Schroll and Munck, 1981; Aboyans et al., 2012). All subjects included in the study (patients and controls) were non-smokers and none of them were taking any medications that had a relationship with the cardiovascular system in the past three months.

The data collection experiments were performed as follows: all participants have been instructed to walk at a self-natural pace in a 50 m indoor corridor and to turn around when they reach the end of the corridor. Experiments involving control volunteers lasted 10 min. Experiments involving PAD patients also had a 10 min limit duration, with the exception of participants that had to stop the walk because of their condition. In the latter case, the experiments lasted as long as possible. The average duration of the experiments involving PAD patients was of 5.75 (± 3.03) min and only two participants reached the 10 min mark.

### 4.2. Analysis setup

We analyzed acceleration signals for each subject. Every sensor recorded three acceleration signals (one per axis), thus obtaining 12 raw signals (4 sensors per subject). Every acceleration signals is analyzed using full spectra analysis, as described in section 3. This analysis was performed in the 0.3 to 15 Hz frequency band.

The differences between healthy and condition groups, as characterized by the spectral indices, were assessed using a non-parametric bootstrap hypothesis test. The null hypothesis (*H*_0_) represented no differences between groups, against the alternative hypothesis (*H*_1_), which represented the existence of significant differences. The model proposed can be stated as follows:

$$\begin{array}{cc} H_{0}: & \mu_{x}=\mu_{y}\\ H_{1}: & \mu_{x}>\mu_{y} \end{array}$$

where μ_*x*_ and μ_*y*_ represented the population mean of a given spectral index for healthy (*X*) and condition (*Y*) group. The bootstrap procedure is based on random sampling with replacement from the available data set. Assuming the model of the reality proposed, (i.e., *H*_0_ is true), a pooled population (*X*∪*Y*) was constructed, formed by healthy and condition subjects. The pooled population reproduces the model of the reality where *H*_0_ holds, and random samples are drawn from this set. The bootstrap hypothesis test aims at estimating the probability of obtaining an observed mean difference $\overset{-}{Z}=\overset{-}{X}-Ȳ$ given *H*_0_, and could be described in the following steps: (1) Two bootstrap random samples, $X_{i}^{*}$ and $Y_{i}^{*}$ are drawn with replacement from *X*∪*Y*. $X_{i}^{*}$ represents the healthy and $Y_{i}^{*}$ represents the PAD groups, so that they are of the same size as the original groups. (2) The bootstrap mean difference is computed as $\overset{-}{Z_{i}^{*}}=\overset{-}{X_{i}^{*}}-\overset{-}{Y_{i}^{*}}$. (3) Steps 1-2 are repeated B times (in this work B = 2000) yielding B different bootstrap mean differences $Z_{1}^{*},\ldots ,Z_{B}^{*}$. Finally, (4) the probability of getting values larger than the observed value is computed to get a p-value

(3) $$\begin{array}{l} p_{Boot}=Pr\left(Z^{*}\geq Z|H_{0}\right)<0.05 \end{array}$$

This probability can be estimated as the fraction of values $Z_{i}^{*}$, larger than the observed value *Z*:

(4) $$\begin{array}{l} {\hat{p}}_{Boot}=\frac{\#\left\{Z_{i}^{*}\geq Z\right\}}{B} \end{array}$$

The *p*-value estimates the probability of obtaining a more extreme value than the observed value *Z*, given the model of the world proposed, i.e., *H*_0_. A *p*-value lower than 0.05 implies that the probability of obtaining such observed value *Z* is so small that it is likely that the proposed model is wrong, then we need to reject *H*_0_.

We also studied the differences between PAD patients with IC on both lower limbs (4 patients) and with IC only on the right lower limb (3 patients). In order to compare both groups, since there are very few patients, mean and standard deviation for each index is estimated using bootstrap resampling, which provides a more robust way to compare results.

## 5. Results

**Figure 4:**
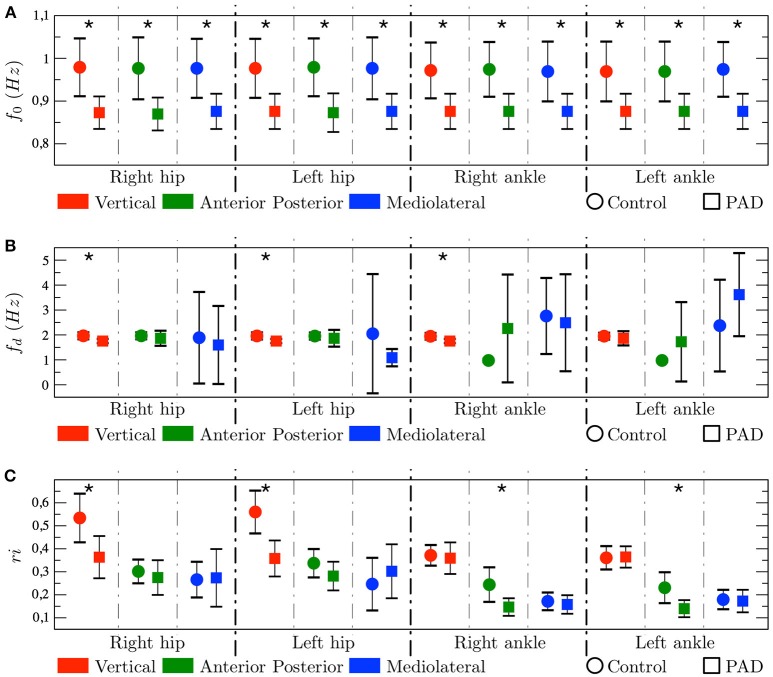
Results represented as mean±std. In each graph, the symbol ^*^ indicates that the *p*-value < 0.05. Tow-tailed test. **(A)** Fundamental frequency, **(B)** Dominant frequency, and **(C)** Regularity index.

Averaging between all sensor locations and axis reveals that the *f*_0_ is statistically higher (*p* < 0.05) in control subjects (0.9743 ± 0.0716) than in PAD patients (0.8748 ± 0.0438). In more detail, Figure 4A shows comparison of *f*_0_ parameter between control (circles) and PAD (squares) subjects. Control subjects had higher *f*_0_, which indicates that the gating frequency is higher (periodicity is lower). Differences between the two groups are indeed statistically significant for all possible comparisons, namely, the three axes for both ankles and both hips.

On the other hand, Figure 4B shows that *f*_*d*_ is, only, different statistically for X-axis in both hips and right ankle. Furthermore, *f*_*d*_ is not always higher for controls. It does not reflect the gating frequency but a harmonic of the gating frequency, in many cases the first harmonic but sometimes higher harmonics, therefore this parameter shows high instability, as represented by the large whiskers.

Figure 4C shows that, *ri* presents, in general, higher values for controls than for PAD subjects, i.e., the spectral energy is more concentrated around the *f*_*d*_ for controls. Interestingly this difference is statistically significant in different axes depending on the node location, namely, in the X-axis (V acceleration) on the hip, and the Y-axis (AP acceleration) on the ankle.

**Figure 5:**
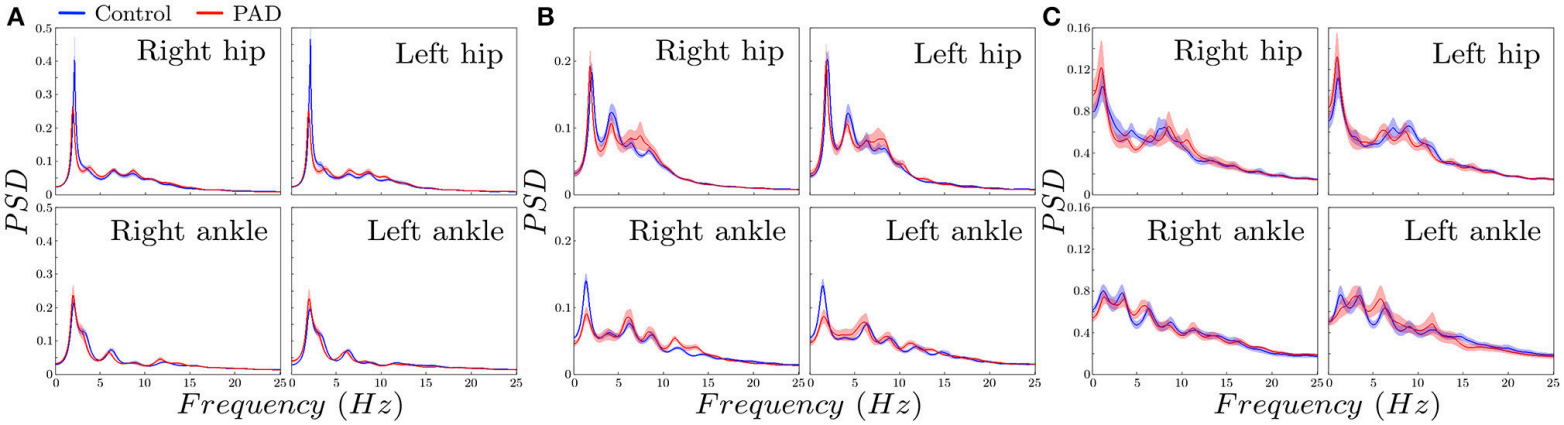
Spectral envelopes estimated with LPC (mean±std using bootstrap) for control subjects and PAD patients. **(A)** vertical, **(B)** Anterior posterior, **(C)** Mediolateral.

Figure 5 shows the spectral envelopes, for controls the spectral energy is always higher for every frequency in the band of interest. Also, the energy is proportionally more concentrated around the *f*_*d*_, specially in the X-axis on the hip, and the Y-axis on the ankle, which is in agreement with the results yielded by *ri* parameter.

**Figure 6:**
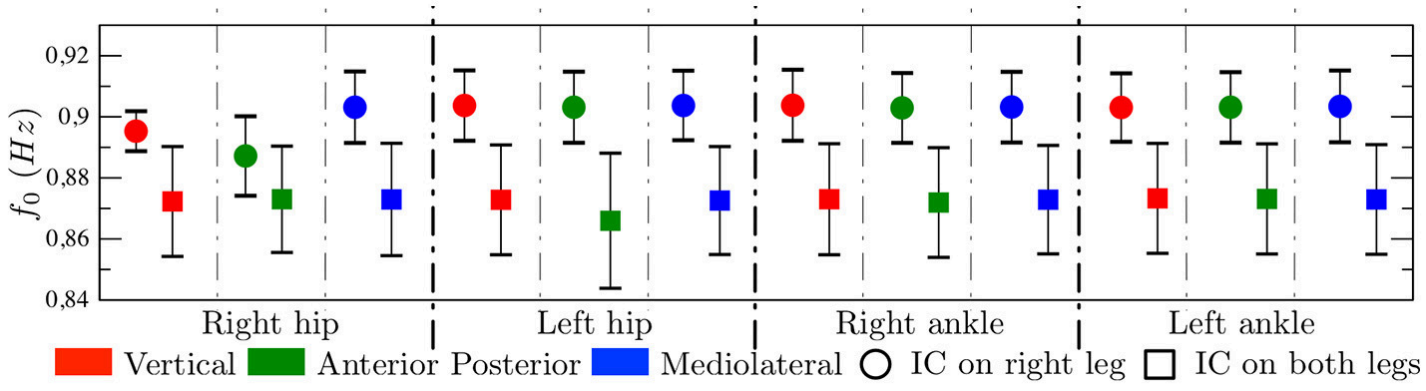
Fundamental frequency represented as mean±std. Comparison between PAD patients with IC on right leg and with IC on both legs. Quantitative results but not hypothesis test.

A few PAD patients present IC in both legs (4) while others in one leg (3 on the right leg and 1 on the left leg). Figure 6 shows that *f*_0_ is in mean higher for patients showing IC in both legs. Due to the small number of subjects in each group we did not perform a statistical analysis.

## 6. Conclusions

In this work we studied the gait acceleration signal, from PAD patients and controls. The data was acquired using four sensors located at hip and ankle height that measured the tri-axial acceleration. The data acquisition equipment allowed natural gait in contrast to traditional treadmill equipment. In this work, we propose a full spectral analysis aiming to characterize both the periodicity (with *f*_0_) and pattern morphology (with *ri*, *bw*_*f*_*d*__, *bw*_*f*_0__, and the spectral envelope) of acceleration gait signals. To that end we analyzed different spectral parameters.

We found significant differences between controls and PAD patients in *f*_0_, for every sensor location and for the three axes. This parameter was always higher for controls, indicating a higher gating frequency. This is possibly due to fear to pain and fall experimented by PAD patients. Regarding *f*_*d*_, differences are only statistically significant for the V acceleration in both hips and right ankle. Furthermore, *f*_*d*_ is not always higher for controls. However, *f*_*d*_ does not reflect the gating frequency but a harmonic of the gating frequency. Therefore, we propose to use *f*_0_ rather than *f*_*d*_ to characterize gait acceleration signals.

Both *ri* and spectral envelopes indicated higher energy concentration around *f*_*d*_ for controls than for PAD patients. Interestingly this difference is statistically significant in different axes depending on the sensor location, namely, in the V acceleration on the hip, and the AP acceleration on the ankle.

We also found, as a preliminary result, that patients presenting IC in both legs may show lower *f*_0_ than patients showing IC in a single leg. This results is consistent with the results of *f*_0_ comparing controls and patients, since IC in both legs can be considered a more severe condition than IC in a single leg.

From a clinical point of view, a full spectral analysis of acceleration gait signals shows a significant decrease in *f*_0_ that allows identifying patients with PAD who present claudication. This difference is observed for every sensor location and for the three axes. The fact that *f*_0_ is lower when the claudication affects both legs reinforces its possible clinical usefulness. The full spectral analysis could be used for clinical early diagnosis and also to monitor the disease evolution in PAD patients.

In future works we expect to increase the dataset in order to provide statistical significance to the different degrees of PAD between these two groups. A larger dataset would also eventually allow an interesting analysis comparing subjects with IC in the right leg, versus subjects with IC the left leg.

In conclusion, the aim of the work is to provide new spectral method, using *f*_0_ and a larger frequency band, to better characterize gait patter in PAD patients. The proposed full spectra approach allowed to better discriminate PAD patients and control subjects, and it also showed promising results to assess severity of PAD.

## Ethics statement

This study was carried out in accordance with the recommendations of the Ethics Committee of Rey Juan Carlos University with written informed consent from all subjects. All subjects gave written informed consent in accordance with the Declaration of Helsinki. The protocol was approved by the Ethics Committee of Rey Juan Carlos University.

## Author contributions

MC, ÓB-P, RG-E, and AC participated in the design of the work and in the analysis or interpretation of data for the work. ES participated in the conception and design of the work. ES, AS, BdlCT, and JN participated in acquisition and interpretation of data for the work. All authors participated in manuscript writing and provided approval for publication of the content.

### Conflict of interest statement

The authors declare that the research was conducted in the absence of any commercial or financial relationships that could be construed as a potential conflict of interest.

## Footnotes

^1^Sample acceleration signals and code to reproduce the proposed analysis is provided as supplementary data, and can be accessed on the following link: https://github.com/MihChi/SIMELeD.git
